# cGAS/STING Pathway Mediates Accelerated Intestinal Cell Senescence and SASP After GCR Exposure in Mice

**DOI:** 10.3390/cells14221767

**Published:** 2025-11-11

**Authors:** Santosh Kumar, Kamendra Kumar, Jerry Angdisen, Shubhankar Suman, Bhaskar V. S. Kallakury, Albert J. Fornace

**Affiliations:** 1 Department of Oncology, Lombardi Comprehensive Cancer Center, Georgetown University Medical Center, Research Building, Room E504, 3970 Reservoir Rd., NW, Washington, DC 20057, USA; 2 Department of Pathology, Georgetown University Medical Center, Washington, DC 20057, USA; 3 Department of Biochemistry and Molecular & Cellular Biology, Georgetown University Medical Center, Washington, DC 20057, USA

**Keywords:** senescence, senescence associated secretory phenotype, inflammation, cGAS/STING signaling, DNA damage, intestinal barrier integrity, galactic cosmic radiation

## Abstract

Long-duration space missions expose astronauts to galactic cosmic radiation (GCR), a complex spectrum of high-charge, high-energy (HZE) ions that pose significant risks of chronic tissue injury. To model these effects, we examined intestinal outcomes in wild-type mice 5 months after low-dose (50 cGy) 33-ion mixed-field GCR simulation (GCRsim). GCRsim induced sustained DNA double-strand breaks (DSBs) and oxidative stress, as shown by elevated γH2AX foci and 4-HNE staining. Intestinal epithelial cells (IECs) exhibited pronounced senescence, marked by increased SA-β-gal activity, p16 upregulation, LaminB1 loss, and induction of senescence-associated secretory phenotype (SASP) cytokines (*Cxcl10*, *IL-6*, *IL-1β*, *Icam1*). GCRsim also elevated circulating LINE-1 DNA and reduced expression of DNA-degrading nucleases (DNase2, TREX1), indicating impaired extracellular DNA clearance. Targeted molecular study revealed persistent activation of the cGAS–STING pathway, with elevated cGAS, STING, pTBK1, pIKKα/β, and nuclear pIRF3, pIRF7, and p65, consistent with chronic innate immune signaling. Functionally, GCRsim altered nutrient absorption gene expression—upregulating glucose transporters (*Slc2a2*, *Slc2a5*, *Slc5a1*) and gut hormones (*Cck*, *Gip*), while downregulating cholesterol/fat transporters (*Npc1*, *Npc1l1*). Biochemical markers supported intestinal injury, with decreased serum citrulline and increased intestinal fatty acid-binding protein (I-FABP), indicating barrier compromise. Collectively, these findings demonstrate that GCRsim drives sustained intestinal dysfunction, highlighting the need for countermeasures to protect GI health during deep-space missions.

## 1. Introduction

Long-duration space missions beyond low Earth orbit, such as those to Mars, expose astronauts to galactic cosmic radiation (GCR), a complex, mixed field of high-charge, high-energy (HZE) ions spanning a range of low- and high-linear energy transfer (LET) particles [1,2,3]. Unlike terrestrial low-LET photon radiation (X-rays or γ-rays), GCR consists of densely ionizing particles capable of producing complex DNA damage, oxidative stress, and persistent tissue dysfunction [4,5,6,7,8]. The gastrointestinal (GI) tract, with its high proliferative rate and continuous epithelial turnover, is particularly vulnerable to such insults, placing barrier integrity, nutrient absorption, and overall intestinal homeostasis at risk [7,8]. Understanding the chronic effects of space radiation on the intestine is therefore critical for evaluating astronaut health risks and developing targeted countermeasures. Most existing in vivo studies have focused on single-ion exposures, such as ^56^Fe or ^28^Si, revealing persistent DNA damage, impaired intestinal epithelial cell (IECs) migration, cellular senescence, senescence-associated secretory phenotype (SASP) activation, and long-term alterations in epithelial cell proliferation and tumorigenesis [7,8,9,10]. While these studies provide important mechanistic insights, they do not fully capture the mixed-field complexity of GCR encountered in space. Exposure to a combination of ions with varying radiation qualities may induce distinct, cumulative, or synergistic patterns of DNA damage, oxidative stress, and tissue responses. Consequently, ground-based in vivo studies using simulated galactic cosmic radiation (GCRsim) is expected to provide a more physiologically relevant model for predicting intestinal injury and functional decline among astronauts than single-ion exposures alone.

Cellular damage from radiation is largely mediated by reactive oxygen species (ROS), which induce oxidative stress by disrupting the balance between pro-oxidants and antioxidants [11]. This imbalance damages DNA, proteins, and lipids, undermining cellular function [12]. Prolonged oxidative stress can trigger cellular senescence, a state of permanent growth arrest accompanied by the SASP, which releases pro-inflammatory cytokines, chemokines, growth factors, and proteases. SASP further exacerbates tissue injury by promoting chronic inflammation and affecting neighboring healthy cells, contributing to degenerative changes and age-related pathologies, including radiation-induced GI injury [7,8,9,13]. Emerging evidence also highlights the role of endogenous stress signals such as LINE-1 retrotransposons, normally silenced repetitive DNA elements, in mediating radiation responses [14,15]. Stressors like DNA damage and oxidative stress can reactivate LINE-1, producing cytosolic DNA that is sensed by the cGAS-STING pathway with potential role in pro-inflammatory cytokine production, amplifying SASP and chronic tissue inflammation [16,17]. In the context of GI injury, these molecular cascades represent key drivers of long-term dysfunction.

In this study, we investigated the chronic intestinal effects of 50 cGy 33-ion GCRsim in wild-type C57BL/6 mice, 5 months post-irradiation. We observed persistent DNA double-strand breaks (DSBs), oxidative stress, and pronounced epithelial senescence, accompanied by robust SASP cytokine induction. GCRsim also elevated circulating LINE-1 DNA, reduced expression of DNA-degrading nucleases, and triggered sustained activation of the cGAS–STING pathway, indicating chronic innate immune activation. Functionally, GCRsim altered nutrient transporter and gut hormone gene expression, and biomarkers of epithelial injury such as serum citrulline and intestinal fatty acid-binding protein (I-FABP) were disrupted, reflecting compromised barrier integrity and intestinal dysfunction. Collectively, these findings demonstrate that low-dose GCRsim induces persistent DNA damage, oxidative stress, senescence, chronic inflammatory signaling, and functional decline in the intestine. The distinct and more severe effects of GCRsim compared to γ-irradiation underscore the biological impact of densely ionizing space radiation and highlight the urgent need for targeted countermeasures to protect GI health during long-duration deep-space missions.

## 2. Materials and Methods

### 2.1. Animal Irradiation and Biospecimen Collection

Male C57BL/6 mice (8–10 weeks old; Jackson Laboratories, Bar Harbor, MA, USA) were shipped to the Brookhaven National Laboratory (BNL) animal facility and acclimated for 5–7 days prior to irradiation. Mice were then exposed to an acute dose of 50 cGy of full-spectrum, 33-ion simulated galactic cosmic radiation (GCRsim) at the NASA Space Radiation Laboratory (NSRL), BNL, Upton, NY, as previously described [18]. The GCRsim includes seven distinct ion species and 14 energy levels of hydrogen and helium, delivered via 33 individual beams in a single exposure (Table S1) [3,19,20]. For comparison, a separate cohort received 50 cGy of γ-radiation using a ^137^Cs source. Following exposure, all mice were transported the next day to Georgetown University (GU) in a temperature-controlled environment to minimize transport-related stress. Mice at both BNL and GU were housed under standardized conditions: temperature- and air-controlled rooms maintained at 22 °C with 50% humidity and a 12 h light/dark cycle. Animals had ad libitum access to food and filtered water throughout the study. Five months post-irradiation, mice were euthanized using a carbon dioxide chamber equipped with a flow-rate controller. Blood and intestinal tissues were collected. The jejunum was chosen for this study due to its high sensitivity to radiation-induced injury and its critical role in nutrient absorption and intestinal immune regulation. As a region of the small intestine characterized by a rapidly renewing epithelial lining and dense population of proliferative crypt cells, the jejunum is particularly vulnerable to radiation-induced DNA damage, oxidative stress, and inflammatory responses [7]. The jejunum was divided into three portions for downstream applications: crypt cell isolation, formalin fixation for histological analysis, and flash-freezing for molecular assays at −80 °C. All procedures were conducted in accordance with the Guide for the Care and Use of Laboratory Animals and approved by the Institutional Animal Care and Use Committees at BNL (Protocol# 345, approved 12 October 2021) and GU (Protocol# 2016-1129, approved 1 August 2021).

### 2.2. Crypt Cells Isolation and Senescent Cell Detection

IECs were isolated as previously described [13]. Briefly, jejunal segments were inverted to expose the lumen, cut into ~10 mm pieces, and thoroughly rinsed with ice-cold phosphate-buffered saline (PBS). The tissues (n = 6) were incubated in 2 mM EDTA in PBS for 20 min at 4 °C to facilitate epithelial cell detachment, followed by two vigorous washes with PBS. The resulting supernatant containing villus cells was discarded. Remaining tissues were incubated with a tissue dissociation solution containing 1 mg/mL STEMxyme^®^ 2 Collagenase/Neutral Protease (Dispase; Cat# LS004112, Worthington Biochemical Corp., Lakewood, NJ, USA) and 0.1 mg/mL DNase I (Cat# 89836, Thermo Scientific, Waltham, MA, USA) in Hank’s Balanced Salt Solution (HBSS; Cat# 14175103, Thermo Scientific) for 20 min at 37 °C. After incubation, the supernatant was discarded, and epithelial cells were released by vigorous shaking in cold HBSS. The cell suspension was passed through a 70 μm cell strainer, centrifuged, washed twice with cold HBSS, and resuspended in HBSS containing 2% fetal bovine serum (FBS). Senescent cells were detected using C12FDG (5-Dodecanoylaminofluorescein Di-β-D-Galactopyranoside; Invitrogen™ Cat# D2893), following the manufacturer’s protocol. Cells were analyzed on a BD LSRFortessa flow cytometer (Becton Dickinson, San Jose, CA, USA) using FACSDIVA software (v9.0). Forward and side scatter parameters were used to exclude debris and doublets. Viable cells were identified by negative staining with SYTOX™ Blue (Cat# S34857, Life Technologies, Frederick, MD, USA), using a 405 nm laser and a 450/40 bandpass filter. Fluorescence signals from C12FDG were collected using excitation/emission settings of 490/514 nm.

### 2.3. Immunostaining

Paraffin-embedded jejunal tissue sections (n = 5) were first deparaffinized, rehydrated through a graded alcohol series, and subjected to antigen retrieval by boiling in pH 6.0 citrate buffer (Electron Microscopy Sciences, Hatfield, PA, USA). For immunohistochemistry, sections were incubated overnight at 4 °C with primary antibodies targeting 4HNE (4-hydroxy-2-nonenal), CXCL10, TREX1, cGAS, STING, phospho-IRF3 (Ser385), phospho-IRF7 (Ser471, Ser472), and phospho-p65 (Ser311). Details of the antibodies used for immunostaining analysis are provided in Table S3. Detection was performed using the Mouse- and Rabbit-Specific HRP/DAB IHC Detection Kit (Cat# AB236466; Abcam, Cambridge, MA, USA) following the manufacturer’s instructions. Slides were counterstained with hematoxylin, dehydrated, and mounted using Permount mounting medium (Cat# SP15-100, Fisher Chemical, Waltham, MA, USA). Brightfield images were acquired using an Olympus microscope. For immunofluorescence analysis, tissue sections were incubated overnight at 4 °C with primary antibodies against Phospho-Histone H2AX (S139) or Lamin B1. After washing, sections were treated with Alexa Fluor-conjugated secondary antibodies—either 488 (green) or 594 (red)—for 1 h at room temperature. Nuclei were counterstained with DAPI. Fluorescent images were captured using an Olympus fluorescence microscope.

### 2.4. Immunoblot Analysis

Frozen intestinal tissues from wild-type mice (n = 5) were processed as described previously [7]. Briefly, samples were lysed in RIPA buffer containing protease and phosphatase inhibitor cocktail (Sigma Aldrich, St. Louis, MO, USA). Equal amount of protein in each group was resolved in SDS PAGE, transferred onto polyvinylidene fluoride (PVDF) membrane, blocked with 5% non-fat milk in tris-buffered saline with 0.1% Tween, and incubated overnight with specific primary antibodies [anti-Lamin B1, anti-p21, anti-IL-1β, anti-β-Tubulin, anti-DNase 2, anti-TREX1, anti-STING, anti-TBK1, anti-IKKα, anti-Phospho-TBK1 (Ser172), ant-Phospho-IKKα/β (Ser176/180) (16A6)]. Details of the antibodies used for immunoblot analysis are provided in Table S3. Immunoblots were developed using HRP-conjugated secondary antibodies and enhanced chemiluminescence (ECL) detection system (Cat# 34080, Thermo Fisher Scientific). The captured images were densitometrically analyzed using ImageJ2 software Fiji version: 2.14.0/1.54f. The mean values from all replicates are presented as fold change relative to the control.

### 2.5. qPCR Assays and Serum DNA Detection

Total RNA was extracted from isolated crypt cells (n = 5) using the RNeasy Mini Kit (Qiagen, Valencia, CA, USA) and then reverse transcribed to cDNA using the RT2 First Strand Kit (Qiagen). Real-time PCR for multiple segments of LINE-1 elements was performed on a CFX96 instrument (Bio-Rad, Hercules, CA, USA), using specific primers (Table S2) and SsoAdvanced Universal SYBR Green Supermix (Bio-Rad, Hercules, CA, USA). The thermocycling protocol included a pre-cycling heat activation at 98 °C for 3 min, followed by 40 cycles of denaturation at 98 °C for 10 s, and annealing and extension at 60 °C for 30 s. All the qPCR reactions were performed in technical triplicates. Gene expression data were analyzed using the ΔΔCt method, with *Gapdh* serving as the reference gene. The final data are presented as fold change relative to the control ± standard error of the mean (SEM). Separately, circulating DNA was extracted from 200 μL of serum using the QIAamp DNA Blood Mini Kit (Qiagen, Valencia, CA, USA). The relative concentration of this circulating serum DNA was determined by qPCR using primers targeting the open reading frames of the mouse LINE-1 element (Table S2). For this, triplicate qPCR reactions were run in 20 μL volumes, each containing the same SsoAdvanced Supermix and 2.5 mM oligos. The real-time PCR amplification was performed on the same CFX96 instrument (Bio-Rad, Hercules, CA, USA), using the same thermocycling conditions as described above. Serum DNA data were analyzed using the ΔΔCt method, with Actnβ serving as the reference DNA as described earlier [21,22].

### 2.6. Barrier Function Analysis

ELISA was performed for detecting Serum (n = 4) intestinal fatty acid binding protein (I-FABP) and serum citrulline. Serum I-FABP concentrations in control and radiation exposed mice (n = 4/group) were measured using Mouse Intestinal Fatty Acid Binding Protein (I-FABP) ELISA kit (Cat# MBS760745, MyBioSource, San Diego, CA, USA). This sandwich-ELISA assay was performed in triplicates, and the assay has a detection limit of 0.1 ng/mL with linear detection range of 0.16 to 10 ng/mL. Serum citrulline levels in control and radiation exposed mice (n = 6/group) were measured using Mouse Citrulline (CIT) ELISA kit (Cat#MBS027373, MyBioSource, San Diego, CA, USA). This quantitative sandwich-ELISA assay was performed in triplicates, and the assay has a detection limit of 0.1 μg/mL with linear detection range of 0.25 to 8 μg/mL.

### 2.7. Imaging, Quantification, and Statistical Analysis

To validate the specificity of immunostaining, appropriate control samples were processed alongside experimental specimens. In each tissue section, random fields of crypt base of mucosa were imaged using cellSens Entry v1.15 (Olympus Corp., Center Valley, PA, USA) for both immunohistochemistry and immunofluorescence. For each marker, the average DAB pixel density or fluorescence intensity was quantified from 10–15 images using ImageJ2 software fiji version: 2.14.0/1.54f as described previously [23]. Statistical analysis was performed using Student’s *t*-test, and one-way analysis of variance (ANOVA), as appropriate, with Tukey’s post hoc tests using SigmaPlot 14.0 (Systat Software Inc., San Jose, CA, USA). Experimental data are presented as the mean ± standard error of the mean (SEM). A *p*-value lower than 0.05 was considered statistically significant.

## 3. Results

### 3.1. GCRsim Induces DNA Double-Strand Breaks and Enhances Lipid Peroxidation

**Figure 1:**
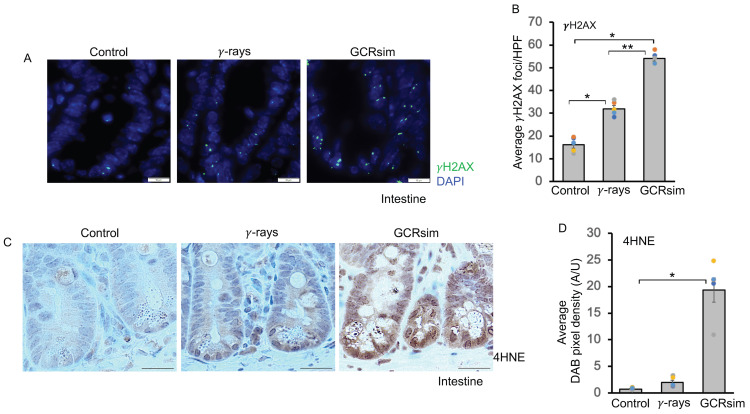
GCRsim induces DNA DSBs and enhances lipid peroxidation in C57BL/6J mouse intestine 150 days after irradiation. (**A**) Representative immunofluorescence images of γH2AX in mouse intestine. γH2AX foci (bright green dots) indicate DNA DSBs. The blue color represents nuclear DAPI staining. Scale bar: 10 μm. (**B**) Quantification of average γH2AX foci in mouse intestine. This bar graph shows the average number of γH2AX foci per field of view (FOV) after irradiation. Data are presented as mean ± SEM. A one-way ANOVA test with Tukey’s comparison post-test was performed. Statistical significance is indicated as follows: * *p* < 0.001, versus the control, ** *p* < 0.001 versus the γ-ray sample. (**C**) Representative images of 4HNE immunostaining in mouse intestine. The images show lipid peroxidation after radiation exposure, with 4HNE staining visible in brown. The counterstain is hematoxylin (blue). Scale bar: 20 μm. (**D**) Quantification of 4HNE expression in mouse intestine. The bar graph represents the average 4HNE DAB pixel density, which is a measure of protein expression. The data demonstrate higher expression in the GCRsim group compared to the γ-ray or control groups. Error bars represent mean ± SEM. A one-way ANOVA test with Tukey’s comparison post-test was performed. Statistical significance is indicated as follows: * *p* < 0.001 versus the control sample.

Immunostaining of intestinal tissue sections for γH2AX, a biomarker of DNA DSBs, revealed significantly higher γH2AX foci in the irradiated group compared to controls (Figure 1A,B). These findings indicate the presence of DNA damage and persistent oxidative stress in intestinal cells following GCRsim exposure. To further confirm oxidative stress, we performed immunodetection of 4-HNE, a lipid peroxidation product commonly associated with oxidative stress and aging [24,25]. Intestinal tissue sections from irradiated mice showed significantly higher 4-HNE staining compared to controls (Figure 1C). Moreover, 4-HNE staining was significantly greater in GCRsim-exposed mice compared to γ-irradiated mice (Figure 1D). Collectively, these results demonstrate the occurrence of lipid peroxidation and DNA damage response after GCRsim exposure, indicating persistent oxidative stress post-irradiation (Figure 1A,D). Despite notable changes in the intestinal tract following radiation exposure, mice showed no significant differences in body weight or visible signs of distress compared to controls over the 5-month observation period. These findings indicate that, although localized intestinal damage occurred, it did not lead to apparent systemic health decline within the duration of the study.

### 3.2. GCRsim Induces Senescence and Acquisition of Senescence Associated Secretory Phenotype

**Figure 2:**
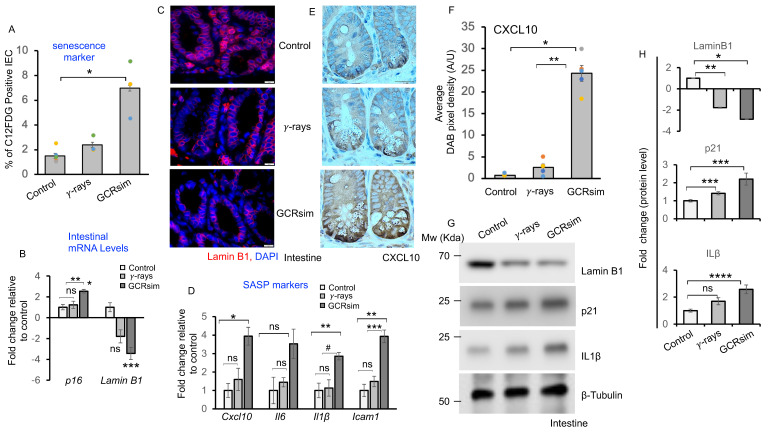
GCRsim induces senescence and acquisition of SASP in mouse intestine. Isolated intestinal epithelial cells were used to detect senescent cells using C12FDG in a flow cytometer. (**A**) Bar graph showing the percentage of C12FDG positive cells showing highest senescent cell frequency in GCRsim-exposed mice relative to γ-ray or control group. A one-way ANOVA test with Tukey’s comparison post-test was performed. Statistical significance is indicated as follows: * *p* < 0.001 versus the control sample (**B**) Relative mRNA expression of senescence markers in the mouse intestinal tissue. Increased p16 and reduced LaminB1 mRNA expression in GCRsim exposed mouse intestinal tissue indicated induction of cellular senescence, relative to control group (n = 5, error bars represent SEM, non-significant (ns) * *p* < 0.001, *** *p* = 0.007, two-sided, versus control, ** *p* = 0.0015 versus γ-ray sample, unpaired t-test). (**C**) Representative immunofluorescence images of LaminB1 showing reduced nuclear expression of LaminB1 in mouse intestine after irradiation. The loss of LaminB1 is a hallmark of senescence. Scale bar: 10 μm. (**D**) qPCR analysis of SASP markers (*Cxcl10*, *IL-6*, *IL-1β*, and *Icam1*) showing fold changes in mRNA levels in mouse intestine after radiation exposure, expressed relative to control (n = 5, error bars represent SEM, non-significant (ns), * *p* < 0.047, ** *p* = 0.006 versus control, # *p* = 0.005, *** *p* = 0.013 versus γ-ray sample, *** *p* = 0.007, two-sided, unpaired *t*-test) (**E**) Representative immunohistochemical images of CXCL10 staining in mouse intestine. Scale bar: 20 μm. (**F**) Quantification of CXCL10 DAB pixel density in the crypt base cells and, presented as a bar graph. A one-way ANOVA test with Tukey’s comparison post-test was performed. Error bars represent mean ± SEM. Statistical significance: * *p* < 0.001, versus the control, ** *p* < 0.001 versus γ-ray sample (**G**) Immunoblot analysis of proteins associated with senescence and inflammation, including LaminB1, p21, and IL-1β, following radiation exposure. (**H**) Mean pixel densities obtained from immunoblot analyses of five independent biological replicates were normalized to β-Tubulin and are presented as fold change relative to the control group (n = 5, error bars represent SEM, non-significant (ns), * *p* < 0.001, ** *p* < 0.002, *** *p* < 0.023, **** *p* = 0.013 versus control, two-sided, unpaired *t*-test.

Flow cytometry analysis using the β-gal assay revealed significantly higher SA-β-gal fluorescent staining in irradiated IECs compared to controls, with the greatest increase observed after GCRsim exposure. In contrast, the increase in SA-β-gal activity after γ-irradiation was not statistically significant compared to controls (Figure 2A). To further assess senescence, we analyzed mRNA expression of p16 and LaminB1 in IECs. GCRsim exposure induced an approximately two-fold increase in p16 mRNA and a three-fold decrease in LaminB1 mRNA relative to controls (Figure 2B). Immunohistochemical detection of LaminB1 in intestinal tissue sections confirmed reduced expression after irradiation, with a greater reduction observed following GCRsim compared to γ-irradiation (Figure 2C). Inflammatory cytokine IL-1β was also elevated (~2.5-fold) following GCRsim exposure. To evaluate SASP gene expression, we performed qPCR analysis and found significant upregulation of *Cxcl10* (~4-fold), *IL-6* (~3.5-fold), *IL-1β* (~3-fold), and *Icam1* (~4-fold) after GCRsim exposure compared to controls (Figure 2D). In contrast, γ-irradiation induced only nominal changes in these markers (Figure 2D). Immunohistochemical analysis of CXCL10 in intestinal sections further confirmed its upregulation after irradiation, with significantly higher expression in GCRsim-exposed tissues compared to γ-irradiated and control groups (Figure 2E,F). To further confirm these observations, we next performed immunoblot analysis for senescence and SASP markers. LaminB1 protein levels were markedly downregulated (~3-fold), whereas p21 and IL-1β was upregulated (~2-fold) in GCRsim-exposed mice compared to controls (Figure 2G,H).

### 3.3. GCRsim Exposure Increases Circulating LINE-1 DNA and Reduces Nuclease Expression

**Figure 3:**
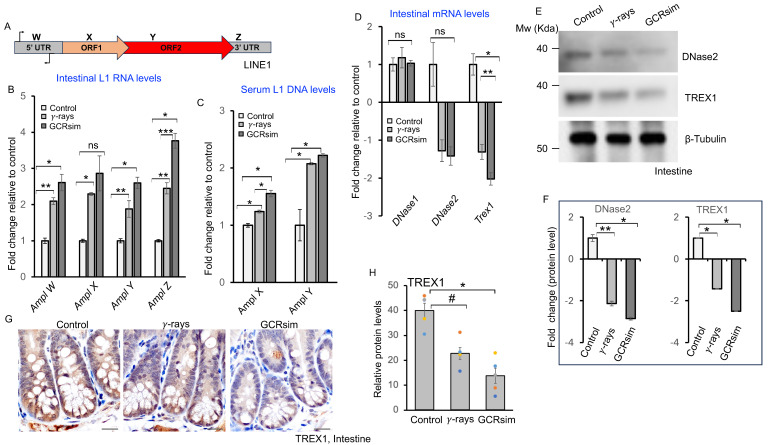
GCRsim induces LINE-1 Activation and Reduces DNA-Degrading Nuclease Expression in Mouse Intestine. (**A**) Schematic representation of the mouse LINE-1 element showing various amplicon. (**B**) qPCR analysis of LINE-1 amplicons in mouse intestine, with bar graph showing increased amplicon levels after irradiation compared to control (n = 5, error bars represent SEM, non-significant (ns), * *p* < 0.001, ** *p* < 0.002, *** *p* = 0.019, two-sided, unpaired *t*-test). (**C**) Detection of extracellular LINE-1 DNA in mouse serum by qPCR, with bar graph showing increased circulating DNA after irradiation (n = 5, error bars represent SEM, * *p* < 0.001, two-sided, unpaired *t*-test). (**D**) qPCR analysis of nuclease genes (*Dnase1*, *Dnase2*, and *Trex1*) in mouse intestine, showing reduced mRNA levels of Dnase2 and Trex1 after irradiation (n = 5, error bars represent SEM, non-significant (ns), * *p* < 0.001, ** *p* = 0.0007, two-sided, unpaired *t*-test). (**E**) Representative immunoblot images showing the expression of DNase2 and TREX1 in mouse intestinal tissue lysates. (**F**) Mean densitometric values from immunoblot analysis of five independent biological replicates were normalized to β-Tubulin and expressed as fold change relative to the control (n = 5, error bars represent SEM, * *p* < 0.001, ** *p* = 0.023, two-sided, unpaired *t*-test). (**G**) Representative immunohistochemical images of TREX1 in mouse intestine. Scale bar: 20 μm. (**H**) Quantification of TREX1 DAB pixel density in the crypt base cells, shown as a bar graph. A one-way ANOVA test with Tukey’s comparison post-test was performed. Error bars represent mean ± SEM. Statistical significance: * *p* < 0.001, # *p* = 0.003 versus the control sample.

Oxidative stress facilitates the release of extracellular DNA (eDNA) into circulation, thereby augmenting the pool of cell-free DNA. Because LINE-1 elements constitute a substantial fraction of cell-free DNA [26,27], we quantified their levels following irradiation. Intestinal tissue analysis revealed a significant increase in LINE-1 element-specific amplicons (Figure 3A) in GCRsim-irradiated mice compared to controls (Figure 3B). Serum analysis confirmed elevated LINE-1 levels, particularly for amplicons X and Y, after GCRsim exposure (Figure 3C), indicating increased circulating DNA content. To investigate mechanisms regulating free DNA levels, we assessed the expression of DNA-degrading enzymes. qPCR analysis showed minimal change in DNase1, but significant downregulation of DNase2 (~1.5-fold) and TREX1 (~2-fold) after GCRsim irradiation (Figure 3D). Immunoblotting further demonstrated marked reductions in DNase2 and TREX1 protein levels in irradiated tissues, corroborated by densitometric analysis (Figure 3E,F). Immunohistochemistry of intestinal tissues confirmed reduced TREX1 staining, with greater suppression after GCRsim than γ-irradiation (Figure 3G,H). Collectively, these findings indicate that low-dose GCRsim exposure increases circulating LINE-1 DNA while suppressing intestinal nuclease expression, suggesting impaired extracellular DNA clearance and altered DNA homeostasis.

### 3.4. GCRsim Exposure Induces Sustained Activation of the cGAS–STING Pathway in the Mouse Intestine

**Figure 4:**
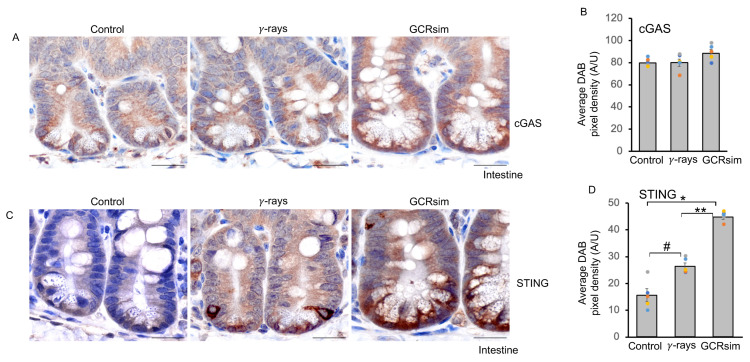
Higher level of cGAS-STING signaling in 50 cGy GCRsim exposed intestine after 150 days. (**A**) Representative immunohistochemical images of cGAS in mouse intestinal tissue sections. (**B**) Quantification of cGAS DAB pixel density, shown as a bar graph. (**C**) Representative immunohistochemical images of STING in mouse intestinal tissue sections. (**D**) Quantification of STING DAB pixel density, shown as a bar graph. Scale bar: 20 μm. A one-way ANOVA test with Tukey’s comparison post-test was performed. Error bars represent mean ± SEM. Statistical significance: * *p* < 0.001, # *p* = 0.002 versus the control sample, ** *p* < 0.001 versus γ-ray sample.

**Figure 5:**
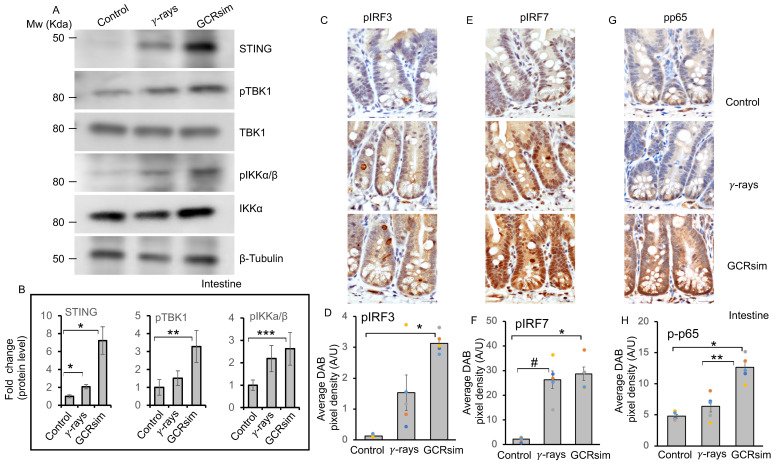
Elevated cGAS–STING downstream signaling in mouse intestine 150 days after 50 cGy GCRsim exposure. (**A**) Representative immunoblot images showing the expression of downstream of the cGAS–STING signaling pathway. (**B**) Band intensity of phosphorylated proteins was normalized to their respective total (non-phosphorylated) protein levels, whereas STING was normalized with β-Tubulin. The bar graph represents fold change in protein expression relative to control, quantified from five independent biological replicates of immunoblot analysis (n = 5, error bars represent SEM, * *p* < 0.005, ** *p* = 0.08, *** *p* = 0.021, two-sided, unpaired *t*-test). (**C**) Representative immunohistochemical images of pIRF3 in mouse intestine. (**D**) Quantification of pIRF3 DAB pixel density, shown as a bar graph. Error bars represent mean ± SEM. Statistical significance: * *p* = 0.004 versus the control sample. (**E**) Representative immunohistochemical images of pIRF7 in mouse intestine. (**F**) Quantification of pIRF7 DAB pixel density. Statistical significance: * *p* < 0.001, # *p* < 0.001 versus control sample. (**G**) Representative immunohistochemical images of phosphorylated p65 (p-p65) in mouse intestine. (**H**) Quantification of p-p65 DAB pixel density. Hematoxylin was used for nuclear counterstaining (blue). Statistical significance: * *p* < 0.001, versus control ** *p* < 0.001 versus the γ-rays. Scale bar 20 μm. A one-way ANOVA test with Tukey’s comparison post-test was performed. Error bars represent mean ± SEM.

Senescence is often associated with aberrant activation of the cGAS–STING signaling axis [28,29,30]. Immunohistochemical analysis revealed uniform cytoplasmic expression of cGAS, whereas STING showed cytoplasmic expression with some individual crypt base cells exhibiting uneven staining. The data showed marginal change in cGAS protein expression, while significant upregulation of STING expression observed in intestinal tissues following irradiation, with greater increases after GCRsim than γ-rays (Figure 4A–D and Figure 5A,B). GCRsim exposure also elevated phosphorylated TBK1 (pTBK1) and phosphorylated IKKα/β (pIKKα/β) levels, indicating robust activation of downstream signaling (Figure 5A). Densitometry confirmed significant fold increases in STING, pTBK1, and pIKKα/β in GCRsim-exposed intestines compared to controls and γ-irradiated samples (Figure 5B). Immunostaining revealed both cytoplasmic and nuclear expression of phosphorylated p65 (p-p65), IRF7 (pIRF7), and IRF3 (pIRF3) in intestinal crypt cells. However, uneven pIRF3 expression was observed in some crypt epithelial cells. The data further demonstrated enhanced nuclear localization of pIRF3, pIRF7, and p-p65 in GCRsim-treated tissues (Figure 5C–H). GCRsim exposure also elevated phosphorylated TBK1 (pTBK1) and phosphorylated IKKα/β (pIKKα/β) levels, indicating robust activation of downstream signaling (Figure 5A). Densitometry confirmed significant fold increases in cGAS, STING, pTBK1, and pIKKα/β in GCRsim-exposed intestines compared to controls and γ-irradiated samples (Figure 5B). Immunohistochemistry further demonstrated enhanced nuclear localization of phosphorylated IRF3 (pIRF3), IRF7 (pIRF7), and p65 (p-p65) in GCRsim-treated tissues (Figure 5C–H). Quantitative pixel density analysis showed these increases were significantly greater than those seen after γ-irradiation. Collectively, these findings indicate that GCRsim induces long-term and potent activation of the cGAS–STING pathway, driving interferon signaling and NF-κB–mediated pro-inflammatory responses. The heightened response to GCRsim relative to γ-rays highlights the unique biological impact of densely ionizing space radiation and underscores the need for targeted countermeasures to mitigate its effects during long-duration space missions.

### 3.5. GCRsim Exposure Perturbs Nutrient Absorption and Intestinal Barrier Integrity in Mouse Gut

**Figure 6:**
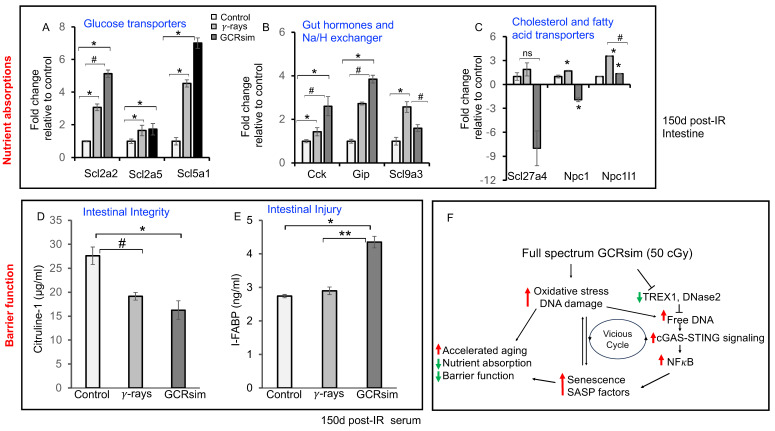
Altered nutrient transporter expression and intestinal integrity markers 150 days after irradiation. (**A**–**C**) Bar graphs showing relative mRNA expression levels of nutrient transporter genes in mouse intestine at 150 days post-irradiation, including (**A**) glucose transporters, (**B**) gut hormones and Na+/H+ exchangers, and (**C**) cholesterol and fatty acid transporters (n = 5, error bars represent SEM, non-significant (ns), * *p* < 0.05, versus control # *p* < 0.05 versus γ-ray sample, two-sided, unpaired *t*-test). (**D**) Serum citrulline levels (μg/mL) measured by ELISA (n = 4). Statistical comparisons were performed using Student’s *t*-test; statistical significance was defined as * *p* < 0.002, # *p* < 0.025 versus control; error bars represent mean ± SEM. (**E**) Serum intestinal fatty acid-binding protein 1 (I-FABP1) levels (ng/mL) measured by ELISA (n = 4). Statistical comparisons were performed using two-sided, unpaired *t*-test; significance was defined as * *p* < 0.001 (verses control) and ** *p* < 0.0015 (versus γ-rays); error bars represent mean ± SEM. (**F**) Schematic illustration of GCRsim-induced events in mouse intestine at long-term time points post-exposure.

At 150 days post-irradiation, qPCR gene expression analysis revealed that acute exposure to 50 cGy GCRsim significantly altered genes regulating nutrient uptake (Figure 6A–C). Glucose transporter genes Slc2a2, Slc2a5, and Slc5a1 were upregulated in GCRsim-exposed intestines compared to controls and γ-ray groups (Figure 6A). Similarly, gut-hormone-related genes (Cck, Gip) and Na+/H+ exchanger-associated transcripts showed increased expression (Figure 6B). In contrast, cholesterol and fatty acid transport genes (Slc27a4, Npc1) were significantly downregulated in the GCRsim group (Figure 6C), suggesting selective disruption of lipid absorption. Markers of intestinal integrity were also altered. Serum citrulline levels were significantly reduced, while intestinal fatty acid-binding protein (I-FABP) levels were elevated in GCRsim-exposed mice compared to both control and γ-ray groups (Figure 6D–E), indicating epithelial injury and barrier compromise. The schematic in Figure 6F summarizes the proposed mechanism: chronic effects of GCRsim-induced oxidative stress and DNA damage activate the cGAS–STING pathway, leading to cellular senescence, SASP release, and NF-κB-mediated inflammation. These processes collectively impair barrier function, disrupt nutrient absorption, and accelerate intestinal aging.

## 4. Discussion

This study provides comprehensive evidence that exposure to GCRsim induces persistent intestinal injury characterized by DNA damage, oxidative stress, cellular senescence, impaired extracellular DNA clearance, activation of the cGAS–STING pathway, and functional decline in epithelial barrier and nutrient absorption. Together, these findings reveal that the intestine is a critical target of chronic space radiation exposure and underscore the distinct and more severe effects of GCRsim compared to conventional γ-irradiation. Our observation of increased γH2AX foci and elevated 4-HNE levels in intestinal tissues demonstrates that mixed-field space radiation induces long-lasting DNA DSB and lipid peroxidation. These results align with prior single-ion studies showing persistent DNA damage in GI and other tissue systems following HZE particle exposure [6,7,8,9,13]. However, the more pronounced oxidative stress seen after GCRsim compared to γ-irradiation highlights the unique biological footprint of high-LET radiation. The persistence of oxidative lesions months after exposure suggests either continuous ROS production or impaired resolution of DNA damage, both of which are linked to accelerated cellular aging and carcinogenesis [6,7,8,13].

The induction of senescence markers (SA-β-gal, p16, LaminB1 loss) and robust upregulation of SASP cytokines (*IL-1β*, *Cxcl10*, *Icam1*) indicate that GCRsim drives a sustained senescence phenotype in intestinal epithelial cells. Importantly, senescence was more pronounced after GCRsim than γ-irradiation, suggesting that high-LET radiation accelerates acquisition of the senescence program. This aligns with models in which radiation-induced oxidative stress (either directly or indirectly via inflammatory signaling) and unresolved DNA lesions act as triggers for senescence, thereby establishing a chronic inflammatory microenvironment. SASP factors such as IL-1β and CXCL10 can reinforce tissue dysfunction by promoting immune cell infiltration, paracrine senescence of neighboring cells, and barrier disruption [31,32]. In the context of deep-space missions, persistent SASP may compromise intestinal homeostasis and contribute to systemic inflammation, increasing the risk of chronic diseases. 

The elevated expression of intestinal LINE1 elements and increased serum DNA levels observed after full-spectrum GCRsim exposure provide strong evidence of persistent genomic instability with systemic consequences. LINE1 retrotransposons, normally repressed under homeostatic conditions, can become mobilized under genotoxic stress, leading to insertional mutagenesis, chromosomal rearrangements, and further genomic disruption [33,34]. Their upregulation in intestinal tissues suggests ongoing DNA destabilization long after the initial radiation insult. In parallel, elevated serum DNA represents a well-established biomarker of cell death, inflammation, and tissue injury [35,36,37], reinforcing the conclusion that GCRsim-induced damage extends beyond local intestinal compartments to systemic circulation. A critical mechanistic link between these findings is the activation of the cGAS–STING pathway, a central sensor of cytosolic DNA and driver of innate immune responses. Cytosolic accumulation of DNA derived from unresolved damage or retrotransposon activity is detected by cGAS, triggering STING-mediated induction of type I interferons and pro-inflammatory cascades [38]. In the context of GCRsim exposure, elevated LINE1 expression and increased circulating DNA likely serve as upstream activators of this pathway. Sustained cGAS–STING activation not only amplifies inflammatory responses through downstream mediators such as NF-κB but may also synergize with SASP factors, thereby creating a feed-forward loop of chronic inflammation and tissue dysfunction. However, other parallel pathways could be activated simultaneously, leading to overlapping downstream outcomes that highlight the complexity of cellular responses, where different molecular routes can compensate for or enhance one another.

The transcriptional and biochemical changes observed in GCRsim-exposed intestines, i.e., upregulation of glucose transporters and gut-hormone-related genes, downregulation of lipid transporters, reduced citrulline, and elevated I-FABP indicate that chronic radiation exposure disrupts nutrient absorption and compromises epithelial barrier function. These alterations are consistent with clinical biomarkers of intestinal injury and provide functional evidence that GCRsim impairs GI physiology beyond molecular and cellular endpoints [7,13]. Together with the observed senescence and inflammatory changes, these findings suggest that the intestine undergoes premature aging-like changes following GCRsim, which may have profound implications for astronaut nutrition, metabolism, and overall health during long-duration missions. Future countermeasure development should therefore prioritize interventions that target oxidative stress, senescence, and innate immune activation, such as senolytics, STING inhibitors, or DNA clearance-enhancing therapies. Moreover, circulating LINE-1 DNA and intestinal biomarkers such as citrulline and I-FABP may serve as non-invasive indicators of radiation-induced intestinal injury, with potential utility in astronaut health monitoring. Collectively, these results suggest that GCRsim-induced GI injury is not restricted to localized epithelial damage but represents a broader systemic stress response driven by genomic instability, extracellular DNA accumulation, and persistent innate immune activation. This chronic cycle of DNA damage, retrotransposon activity, and inflammatory signaling may contribute to long-term health risks in astronauts, including premature tissue aging, metabolic dysfunction, and increased susceptibility to cancer or inflammatory disorders during deep-space missions [39].

Together, these functional deficits are mechanistically linked to persistent DNA damage, cellular senescence, and chronic inflammation induced by GCRsim (Figure 6F), revealing a critical vulnerability of the GI system during spaceflight. While GCRsim studies provide valuable insights into the effects of space radiation, several limitations must be considered when applying these findings to human spaceflight. Animal models, though essential, differ from humans in metabolism, immune function, gut microbiome, and radiation sensitivity, which can affect how results translate [40,41]. The GCRsim setup, despite its sophistication, cannot fully replicate the continuous, multidirectional, and variable nature of galactic cosmic rays encountered in space. Additionally, radiation doses and exposure durations in some studies may not accurately reflect the lower, chronic exposure astronauts experience during long missions [42,43,44]. It should be noted, however, that we and others have not found low-dose-rate sparing effects with high LET radiation [45]. Focusing solely on the GI system overlooks complex interactions with other organs such as the brain, heart, and immune system, which can indirectly influence GI health. Other spaceflight stressors—like microgravity, disrupted circadian rhythms, isolation, and confinement—are not included in these ground-based studies but may synergize with radiation to produce different or more severe effects [46,47]. Many studies track only short-term outcomes, potentially missing late-onset conditions like cancer. Furthermore, individual variability in radiation response—shaped by genetics, diet, and health status—is often not fully captured, limiting generalizability to diverse astronaut populations. Building on these findings, future research should prioritize long-term, longitudinal GCRsim studies to better simulate chronic deep-space exposures and reveal cumulative effects. Additionally, investigations into combined stressors, including microgravity and dietary changes, are essential to reflect true spaceflight conditions [48]. In addition to GCR, several other radiation sources contribute to the complex space radiation environment. Secondary neutrons generated by GCR interactions with spacecraft shielding materials can significantly enhance biological dose and tissue damage due to their high relative biological effectiveness (RBE). Furthermore, sporadic solar proton events (SPEs) can deliver acute, high-dose exposures, while preceding γ-ray bursts (GRBs), though rare, may add further radiation risk depending on mission timing and trajectory [44,49,50]. These additional components should be considered when modeling space radiation exposure and assessing potential health risks for long-duration space travel.

## 5. Conclusions

The collective findings from current study provides a comprehensive overview of the pathophysiological effects of GCRsim on the GI tract. The data illustrate a sequential cascade of events—beginning with DNA damage and oxidative stress, progressing to persistent cellular senescence and chronic inflammation mediated by SASP and cGAS-STING signaling, and culminating in genomic instability, impaired nutrient absorption, and compromised barrier function. Together, these results underscore the significant impact of GCRsim exposure on GI health. Importantly, these insights have critical implications for risk assessment and the development of targeted countermeasures to protect both astronauts undertaking long-duration deep-space missions and patients receiving particle-based radiotherapy from GI-associated radiation injury.

## Data Availability

All data is contained within the article.

## Supplementary Materials

The following supporting information can be downloaded at: https://www.mdpi.com/article/10.3390/cells14221767/s1, Table S1: Full GCR Simulation in order of delivery- comprised of seven different species of ion, and fourteen different energies of H and He for 33 separate beams in a single GCRSim exposure; Table S2: List of primers; Table S3: Antibody details.

## Abbreviations

The following abbreviations are used in this manuscript:

GI	Gastrointestinal
IECs	Intestinal epithelial cells
GCRsim	Galactic cosmic radiation simulation
SPEs	Solar Particle events
GRBs	Gamma ray bursts
HZE	High atomic number and high energy
ROS	Reactive oxygen species
DSBs	DNA double strand breaks
LINE1	Long interspersed nuclear element 1
SASP	Senescence-associated secretory phenotype
cGAS	Cyclic GMP-AMP synthase
STING	Stimulator of Interferon Genes
IRF	Interferon Regulatory Factor
IL	Interleukin
CXCL	Chemokine (C-X-C motif) Ligand
ICAM	Intercellular Adhesion Molecule 1
ELISA	Enzyme-Linked Immunosorbent Assay
