# Supplementary material for: cGAS/STING Pathway Mediates Accelerated Intestinal Cell Senescence and SASP After GCR Exposure in Mice
Source: Cells. 2025 Nov 11;14(22):1767. doi: 10.3390/cells14221767 (PMC12651403; doi:10.3390/cells14221767)
Supplement: Supplementary file 1 [file cells-14-01767-s001.zip › cells-3897976-supplementary.pdf]

Table S1: Full GCR Simulation in order of delivery- comprised of seven different species of ion, and fourteen different energies of H and He for 33 separate beams in a single GCRSim exposure.

S.No.	Z	Ion	Energy (MeV/n)	Fraction
1	1	H	1000	24.71%
2	2	He	1000	4.98%
3	28	Si	600	1.62%
4	1	H	20	6.08%
5	1	H	23	1.34%
6	2	He	20	2.20%
7	2	He	23	0.42%
8	22	Ti	1000	0.90%
9	2	He	27	0.44%
10	2	He	32	0.46%
11	1	H	27	1.48%
12	1	H	32	1.60%
13	1	H	37	1.74%
14	1	H	43	1.86%
15	2	He	37	0.50%
16	2	He	43	0.52%
17	16	O	350	3.08%
18	2	He	50	0.54%
19	2	He	59	0.54%
20	1	H	50	2.00%
21	1	H	59	2.12%
22	1	H	69	2.22%
23	1	H	80	2.24%
24	2	He	69	0.54%
25	2	He	80	0.54%
26	12	C	1000	2.34%
27	2	He	100	1.22%
28	1	H	100	5.44%
29	1	H	150	7.00%
30	2	He	150	1.50%
31	26	Fe	600	0.82%
32	2	He	250	13.77%
33	1	H	250	3.28%

Table S2: List of primers

**Name**	**Forward primer sequence**	**Reverse primer sequence**
Ampl W	5’-CTGCCTTGCAAGAAGAGAGC	5’-AGTGCTGCGTTCTGATGATG
Ampl X	5’-ATCTGTCTCCCAGGTCTGCT	5’-TCCTCCGTTTACCTTTCGCC
Ampl Y	5’-GCTTCGGTGAAGTAGCTGGA	5’-TTCGTTAGAGTCACGCCGAG
Ampl Z	5’-AGCCAAATGGATGGACCTGG	5’-AAGGAGGGGCATAGTGTCCA
Slc2a2	5’-CGAATTTATCCAGCAGCACAAG	5’-GGCCATCAACATGATCTTCAC
Slc2a5	5’-TGATATAGGGCATCCAGGAGAT	5’-TGACCATCCTCACGATCTTTG
Slc5a1	5’-GATAAAAGACCCCACCAGCAT	5’-CTTGGGCCTGGATATATACTTGG
Cck	5’-CCGGTCACTTATTCTATGGCT	5’-ACTGCTAGCGCGATACATC
Gip	5’-GCAAGCTAAGGTCAGAGTCC	5’-GATGATGTGCTGAGAGACCTT
Slc9a3	5’-CCTCATGCTCTGAAGTTCCA	5’-CGGCCAAGCTGTACAAGAG
Slc27a4	5’-CATCCTGTCCTTTGTGTACCC	5’-CATCCTGTCCTTTGTGTACCC
Npc1	5’-CCCTCCAAATTTCGTAAGTGTG	5’-GCAGCCATATAACGAGAGCAT
Npc1/1	5’-GCTAGTTTCTCCTCCAGTACCA	5’-CTCAACCTCCTGATCACCTTG
DNAse2	5’-GCTCAGCTGGGGACTCTAC	5’-GGTCTGGCCGAAGGTTTGA
TREX1	5’-CGTCAACGCTTCGATGACA	5’-AGTCATAGCGGTCACCGTT
DNAse1	5’- CTCGGGGGCTCATCATACAG	5’-TTAGCCCTGTGTACCGCATC
p16	5’-GGGTTTCGCCCAACGCCCCGA	5’-TGCAGCACCACCAGCGTGTCC
Lamin B1	5’-CAGGAATTGGAGGACATGCT	5’-GAAGGGCTTGGAGAGAGCTT
Cxcl10	5’-CCAAGTGCTGCCGTCATTTTC	5’-GGCTCGCAGGGA TGA TTTCAA
Il6	5’-CAAGAAAGACAAAGCCAGAGTC	5’-GAAATTGGGGTAGGAAGGAC
Il1β	5’-GCACTACAGGCTCCGAGATGAAC	5’-TTGTCGTTGCTTGGTTCTCCTTGT
Icam1	5’-GTGGCGGGAAAGTTCCTG	5’-CGTCTTGCAGGTCATCTTAGGAG
Gapdh	5’-CAACTACATGGTCTACATGTTC	5’-CGCCAGTAGACTCCACGAC
ActnB	5’-GTGGTTACAGGAAGTCCCTCAC	5’-CTCCTCTTAGGAGTGGGGGTG

Table S3 Antibody details

S.No.	Antibody	Catalog#	Dilution	Company
1	Phospho-IRF3 (Ser385) Polyclonal Antibody	PA5-36775	IB 1:1000<br>IHC 1:200	ThermoFisher Scientific
2	Phospho-IRF7 (Ser471, Ser472) Polyclonal Antibody	PA5-114592	IB 1:1000<br>IHC 1:200	ThermoFisher Scientific
3	CXCL10 Recombinant Rabbit Monoclonal Antibody (10H11L3)	701225	IHC 1:25	ThermoFisher Scientific
4	Phospho-TBK1 (Ser172) Polyclonal Antibody	PA5-105919	IB 1:1000	ThermoFisher Scientific
5	DNase 2 alpha Polyclonal Antibody	bs-7652R	IB 1:1000<br>IHC 1:200	ThermoFisher Scientific
6	Rabbit Polyclonal TREX1 ANTIBODY	TREX1-101AP	IB 1:500<br>IHC 1:100	Fabgennix Inc

7	cGAS Antibody	orb412701	IB 1:1000<br>IHC 1:200	Biorbyt
8	Rabbit anti-STING/TMEM173 Antibody	orb178801	IB 1:1000<br>IHC 1:200	Biorbyt
9	IRF-3	sc-376455	IB 1:1000	Santa Cruze Biotechnology
10	IRF-7	sc-74471	IB 1:1000	Santa Cruze Biotechnology
11	Lamin B1 (A-11)	sc-377000	IF 1:100	Santa Cruze Biotechnology
12	p-NFκB p65	sc-101748	1:200	Santa Cruze Biotechnology
13	Mouse anti-goat IgG-HRP	sc-2354	IB 1:2500	Santa Cruze Biotechnology
14	Mouse anti-rabbit IgG-HRP	sc-2357	IB 1:2500	Santa Cruze Biotechnology
15	donkey anti-mouse IgG-HRP	sc-2314	IB 1:2500	Santa Cruze Biotechnology
16	TBK1 Antibody (6D603):	sc-73115	1:1000	Santa Cruze Biotechnology
17	Phospho-IKKα/β (Ser176/180) (16A6) Rabbit mAb	2697	IB 1:500	Cell Signaling Technology
18	Anti-p21 antibody	ab188224	IB 1:500	Abcam
19	Anti-4 Hydroxynonenal antibody [HNEJ-2]	ab48506	IHC 1:300	Abcam
20	Mouse IL-1β	AF-401-NA	IB 1:1000	R&D Systems
21	Mouse Phospho- Histone H2AX (S139) Antibody	4418-APC-100	IF 1:50	R&D Systems
